# Early-stage heart failure with preserved ejection fraction in the pig: a cardiovascular magnetic resonance study

**DOI:** 10.1186/s12968-016-0283-9

**Published:** 2016-09-30

**Authors:** Ursula Reiter, Gert Reiter, Martin Manninger, Gabriel Adelsmayr, Julia Schipke, Alessio Alogna, Alexandra Rajces, Aurelien F. Stalder, Andreas Greiser, Christian Mühlfeld, Daniel Scherr, Heiner Post, Burkert Pieske, Michael Fuchsjäger

**Affiliations:** 1 Division of General Radiology, Department of Radiology, Medical University of Graz, Auenbruggerplatz 9/P, 8036 Graz, Austria; 2 Siemens Healthcare, Graz, Austria; 3 Division of Cardiology, Department of Internal Medicine, Medical University of Graz, Graz, Austria; 4 Hannover Medical School, Institute of Functional and Applied Anatomy, Hannover, Germany; 5 Department of Internal Medicine and Cardiology, Campus Virchow Klinikum, Charité University Medicine Berlin, Berlin, Germany; 6 Siemens Healthcare GmBH, Erlangen, Germany; 7 Department of Internal Medicine and Cardiology, German Heart Center Berlin, Berlin, Germany; 8 Berlin Institute of Health, Berlin, Germany

**Keywords:** Diastolic dysfunction, Heart failure with preserved ejection fraction, Cardiovascular magnetic resonance, Dobutamine stress, Porcine model

## Abstract

**Background:**

The hypertensive deoxy-corticosterone acetate (DOCA)-salt-treated pig (hereafter, DOCA pig) was recently introduced as large animal model for early-stage heart failure with preserved ejection fraction (HFpEF). The aim of the present study was to evaluate cardiovascular magnetic resonance (CMR) of DOCA pigs and weight-matched control pigs to characterize ventricular, atrial and myocardial structure and function of this phenotype model.

**Methods:**

Five anesthetized DOCA and seven control pigs underwent 3 T CMR at rest and during dobutamine stress. Left ventricular/atrial (LV/LA) function and myocardial mass (LVMM), strains and torsion were evaluated from (tagged) cine imaging. 4D phase-contrast measurements were used to assess blood flow and peak velocities, including transmitral early-diastolic (E) and myocardial tissue (E’) velocities and coronary sinus blood flow. Myocardial perfusion reserve was estimated from stress-to-rest time-averaged coronary sinus flow. Global native myocardial T1 times were derived from prototype modified Look-Locker inversion-recovery (MOLLI) short-axis T1 maps. After in-vivo measurements, transmural biopsies were collected for stereological evaluation including the volume fractions of interstitium (V_V_(int/LV)) and collagen (V_V_(coll/LV)). Rest, stress, and stress-to-rest differences of cardiac and myocardial parameters in DOCA and control animals were compared by *t*-test.

**Results:**

In DOCA pigs LVMM (*p* < 0.001) and LV wall-thickness (end-systole/end-diastole, *p* = 0.003/*p* = 0.007) were elevated. During stress, increase of LV ejection-fraction and decrease of end-systolic volume accounted for normal contractility reserves in DOCA and control pigs. Rest-to-stress differences of cardiac index (*p* = 0.040) and end-diastolic volume (*p* = 0.042) were documented. Maximal (*p* = 0.042) and minimal (*p* = 0.012) LA volumes in DOCA pigs were elevated at rest; total LA ejection-fraction decreased during stress (*p* = 0.006). E’ was lower in DOCA pigs, corresponding to higher E/E’ at rest (*p* = 0.013) and stress (*p* = 0.026). Myocardial perfusion reserve was reduced in DOCA pigs (*p* = 0.031). T1-times and V_V_(int/LV) did not differ between groups, whereas V_V_(coll/LV) levels were higher in DOCA pigs (*p* = 0.044).

**Conclusions:**

LA enlargement, E’ and E/E’ were the markers that showed the most pronounced differences between DOCA and control pigs at rest. Inadequate increase of myocardial perfusion reserve during stress might represent a metrics for early-stage HFpEF. Myocardial T1 mapping could not detect elevated levels of myocardial collagen in this model.

**Trial registration:**

The study was approved by the local Bioethics Committee of Vienna, Austria (BMWF-66.010/0091-II/3b/2013).

## Background

Heart failure with preserved ejection fraction (HFpEF) is a multifactorial heterogeneous clinical syndrome that is recognized as an independent risk factor for mortality and cardiovascular morbidity [1–3]. Mechanisms leading to symptomatic heart failure with preserved ejection fraction are incompletely understood, challenging the investigation of experimental models at risk to develop this syndrome [4]. Large animal models for HFpEF have been sparsely proposed and studied [5]. The deoxy-corticosterone acetate (DOCA)-salt-induced hypertensive pig (hereafter, DOCA pig) has been introduced as a large animal model for mineralocorticoid-induced hypertension [6–8]. DOCA-salt-induced hypertension in the pig is associated with concentric left ventricular (LV) hypertrophy [9, 10], increased peripheral vascular resistance [11–14], and alterations in the contractile apparatus in vascular smooth muscle cells [15, 16]. Recently Schwarzl at al. [17] documented left atrial (LA) dilatation, normal LV end-diastolic pressure at rest, but leftward shifted end-diastolic pressure–volume relationship, myocyte hypertrophy, titin isoform shift, reduced total-titin phosphorylation in the sub-endocardial layer, and increased LV end-diastolic pressures at lower cardiac output during maximum simulated exercise in DOCA pigs. The authors concluded that this model of hypertensive heart disease mimics the cardiac phenotype of early-stage HFpEF [17, 18]. Imaging parameters of ventricular, atrial and myocardial structure and function were, however, not characterized for this large animal model [17].

Cardiovascular magnetic resonance (CMR) is the standard of reference for non-invasive assessment of cardiac and myocardial function and morphology [19–22]. Employing CMR techniques such as cine imaging, myocardial tagging and 4D flow imaging at rest and during stress as well as myocardial T1 mapping should make it possible to investigate cardiac and myocardial function, evaluate stress-induced differences in cardiac or myocardial function and performance, and identify myocardial tissue alterations in a single investigation in DOCA pigs.

The aim of our explorative study was to evaluate comprehensive CMR imaging of DOCA pigs and weight-matched control pigs at rest and during dobutamine stress to characterize ventricular, atrial and myocardial structure and function of this phenotype model of early-stage HFpEF, and to identify potential non-invasive imaging markers of the disease.

## Methods

The explorative study was approved by the local Bioethics Committee of Vienna, Austria (BMWF-66.010/0091-II/3b/2013) and conformed to the guide for the care and use of laboratory animals, US National Institute of Health (NIH Publication No. 85–23, revised 1996). Thirteen female landrace pigs were enrolled. Arterial hypertension was induced in six animals by subcutaneous implantation of DOCA pellets (100 mg/kg, 90-day release, Innovative Research of America, USA) in combination with a high-salt, high-sugar, high- potassium diet. After 12 weeks of treatment, animals were examined by CMR imaging at rest and during dobutamine stress. One DOCA pig was excluded from analysis because of heart rate and blood pressure instability during the measurements, which could be attributed to florid pericarditis when attempting to acquire histologic samples. Seven weight-matched healthy animals served as controls. The characteristics of the DOCA and the control animals are summarized in Table 1.

**Table 1:** Characteristics of control and DOCA animals

	Controls (*n* = 7)			DOCA (*n* = 5)			Controls vs. DOCA	
Parameter	Rest	Stress	*p*	Rest	Stress	*p*	p_rest_	p_stress_
weight (kg)	58 ± 9			65 ± 2			0.090	
BSA (m^2^)	1.05 ± 0.10			1.14 ± 0.03			0.085	
Htc (%)	28 ± 2			28 ± 2			0.796	
DB (μg · kg^−1^ · min^−1^)		2.7 ± 0.7			3.8 ± 1.0			0.045
HR (min^−1^)	89 ± 5	114 ± 3	< 0.001	86 ± 8	110 ± 13	0.014	0.424	0.488
mBP (mmHg)	87 ± 7	95 ± 13	0.061	106 ± 8	101 ± 12	0.538	0.001	0.400
sBP (mmHg)	103 ± 8	116 ± 9	0.004	125 ± 6	120 ± 11	0.536	< 0.001	0.493
dBP (mmHg)	75 ± 9	82 ± 16	0.077	96 ± 10	86 ± 12	0.122	0.003	0.614
RPP (10^2^ mmHg · min^−1^)	92 ± 8	132 ± 11	< 0.001	108 ± 14	132 ± 17	0.026	0.034	0.990

*BSA* body surface area, *Htc* hematocrit, *DB* dobutamine infusion rate, *HR* heart rate, *mBP* mean blood pressure, *sBP* systolic blood pressure, *dBP* diastolic blood pressure, *RPP (= sBP × HR)* rate-pressure product

*p* is related to the rest-stress comparison within each group. p_rest_ and p_stress_ relate to group comparisons at rest and stress, respectively

### Experimental preparation

Animals were sedated by intramuscular administration of ketamine (20 mg · kg^−1^), midazolam (0.25 mg · kg^−1^) and azaperone (5 mg · kg^−1^). Anesthesia was induced by 30–60 mg propofol (Propofol “Fresenius” 1 %-Emulsion, Fresenius Kabi, Austria) to allow endotracheal intubation. Pigs were mechanically ventilated (Titus, Dräger Medical, Germany) and anesthesia was maintained with sevoflurane (1.5–2.5 %), fentanyl (35 μg · kg^−1^ · h^−1^), midazolam (1.2 mg · kg^−1^ · h^−1^), ketamine (2–8 mg · kg^−1^ · h^−1^) and pancuronium (0.2 mg · kg^−1^ · h^−1^). Respiratory gases (PM 8050 MRI, Dräger Medical, Germany), heart rate and arterial blood pressure (Precess 3160, InVivo, FL, US) were continuously monitored. Sheath accesses of the left internal carotid artery and jugular vein were surgically prepared. Blood samples collected from the arterial line were used to control oximetric and metabolic parameters (ABL700, Radiometer Medical ApS, Denmark). A balanced crystalloid infusion (Elo-Mel Isoton, Fresius Kabi, Austria) was administered at a fixed rate of 10 ml · kg^−1^ · h^−1^ throughout the protocol. Oral temperature of animals was assessed by a sublingual thermometer and was maintained at 38 °C during CMR imaging via air ventilation and/or infusion of cold saline solution.

### Image acquisition

CMR was performed on a 3 T MR scanner (Magnetom Trio, Siemens Healthcare, Germany) using a phased-array 6-channel body matrix coil together with a spine matrix coil. Subjects were investigated in a single session during free breathing in the supine position with electrodes for electrocardiographic (ECG) gating positioned on the chest. After assessment of cardiac and myocardial function, blood flow and LV T1 times at rest, measurements were repeated during stress, which was induced by intravenous infusion of dobutamine (ERWO Pharma, Austria) at rates of 2–5 μg · kg^−1^ · min^−1^, targeting a heart rate increase of approximately 25 %.

**Fig. 1:**
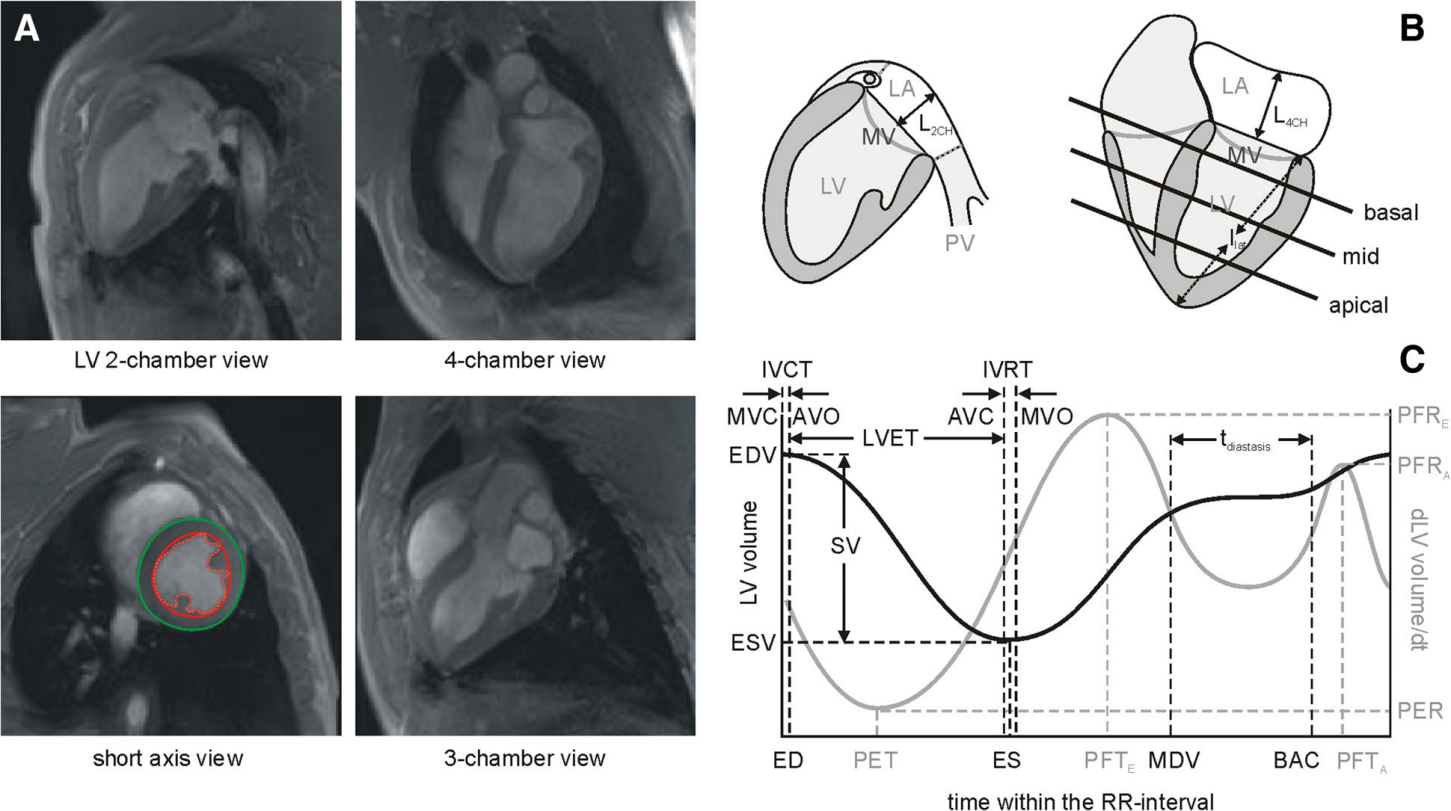
Functional cine images and their evaluation. **a** Diastolic images of cine FLASH series in LV 2-chamber, 4-chamber, 3-chamber and mid-ventricular short-axis views. Subepicardial (*green line*) and subendocardial (*red line*) contouring in short axis images was employed to derive LV volume vs. time curves, wall thickness and left ventricular muscle mass (*red dashed line*). **b** Schematic drawing explaining derivation of left atrial volumes and mitral annular plane systolic excursion (MAPSE). LV = left ventricle; LA = left atrium area (*white plane*); MV = mitral valve; PV = pulmonary vein; L_4CH_, L_2CH_ = length of LA extension in 4-chamber view and 2-chamber view, respectively; l_lat_ = distance from the apex to the lateral mitral annulus. **c** LV volume vs. time curve (*black line*) and its derivative (*gray line*). ED = time of LV end-diastole; ES = time of LV end-systole; MDV = mid-diastolic time after rapid LV filling; BAC = time before atrial contraction; EDV = LV end-diastolic volume; ESV = LV end-systolic volume; SV = LV stroke volume; t_diastasis_ = duration of LV diastasis; PER = peak ejection rate; PET = peak ejection time; PFR_E_ = early diastolic peak filling rate; PFT_E_ = early diastolic peak filling time; PFR_A_ = late diastolic peak filling rate; PFT_A_ = late diastolic peak filling time. Times of aortic valve closure (AVC), aortic valve opening (AVO), mitral valve closure (MVC), and mitral valve opening (MVO) were assessed from cine 3-chamber view series. IVRT = isovolumetric relaxation time; IVCR = isovolumetric contraction time; LVET = left ventricular ejection time

For assessment of ventricular and atrial function, retrospectively ECG-gated, 2D segmented fast low-angle shot (FLASH) cine images (temporal resolution, 27 ms interpolated to 40 cardiac phases; echo time, 2.7 ms; flip angle, 15°-20°; voxel size, 1.9 × 1.6 × 6.0–8.0 mm^3^) were obtained in the LV two-chamber, three- and four-chamber views (Fig. 1), and in contiguous short-axis slices covering the entire LV in 12–14 slices. Two-fold averaging was used to suppress breathing artefacts.

Aortic cross section, vessel wall thickness, and aortic peak blood velocity were evaluated on retrospectively ECG-gated, two-dimensional spoiled gradient-echo-based through-plane velocity encoded cine phase-contrast images (velocity encoding, 110–200 cm · s^−1^; temporal resolution, 30 ms interpolated to 40 cardiac phases; echo time, 2.5 ms; flip angle, 18°; voxel size, 1.8 × 1.6 × 6.0 mm^3^; 2-fold averaging) with image orientation adjusted perpendicular to the course of the proximal ascending aorta 2 cm above the aortic valve. Time-resolved three-directional phase-contrast imaging (4D flow) data were acquired to measure mitral annular tissue velocity and blood flow in the left heart, the pulmonary veins and the coronary sinus; the structures were covered by gapless slices with a retrospectively ECG-gated, two-dimensional spoiled gradient-echo-based three-directional velocity-encoded cine phase-contrast sequence (velocity encoding in all directions, 110 cm · s^−1^; measured temporal resolution, 46 ms interpolated to 25 cardiac phases per cardiac cycle; echo time, 2.9 ms; flip angle, 15°; voxel size, 2.5 × 1.8 × 4.0 mm^3^; 3-fold averaging).

**Fig. 2:**
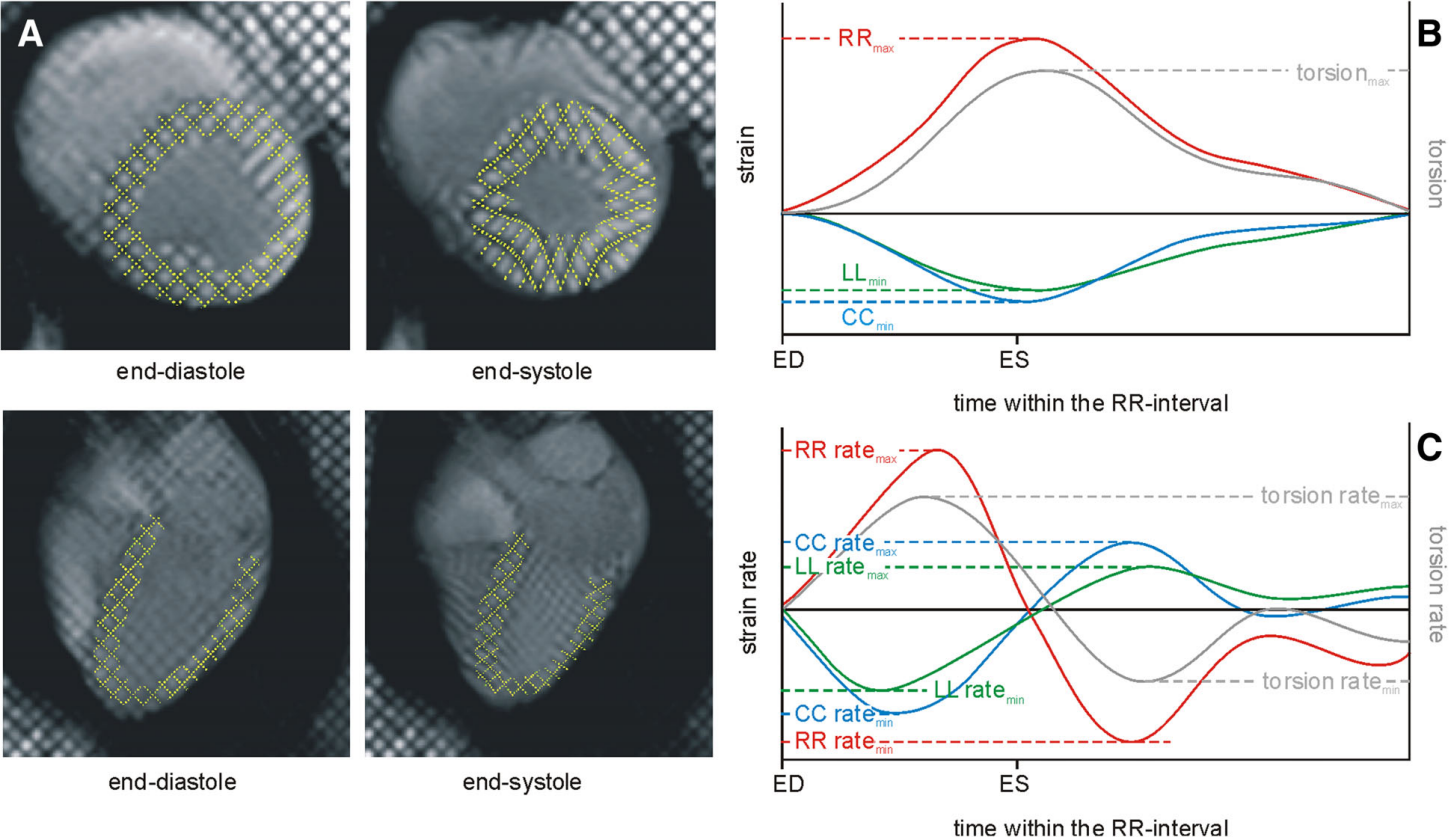
Cine tagged images and their evaluation. **a** End-diastolic and end-systolic images of cine tagging series in mid-ventricular short-axis and 4-chamber orientations. Yellow grid lines detected by the evaluation software are overlaid. **b** Derived time courses of LV myocardial radial (RR, *red line*), circumferential (CC, *blue line*) and longitudinal (LL, *green line*) strains and of LV myocardial torsion (*gray line*). Extrema of curves are annotated. **c** Derived time courses of LV myocardial radial (RR, *red line*), circumferential (CC, *blue line*) and longitudinal (LL, *green line*) strain rates and of LV myocardial torsion rate (*gray line*). Systolic and early diastolic extrema of curves are annotated. ED = time of LV end-diastole; ES = time of LV end-systole

To study myocardial strain, tagged cine images (Fig. 2) were acquired with a retrospectively ECG-gated FLASH with spatial modulation of magnetization (SPAMM) in the short axis (basal, mid-ventricular and apical) and in the 4-chamber orientation (grid spacing, 6 mm; temporal resolution, 20 ms interpolated to 50 cardiac phases; echo time, 3.3 ms; flip angle, 12°; voxel size, 1.8 × 1.3 × 6.0–8.0 mm^3^; 3-fold averaging).

**Fig. 3:**
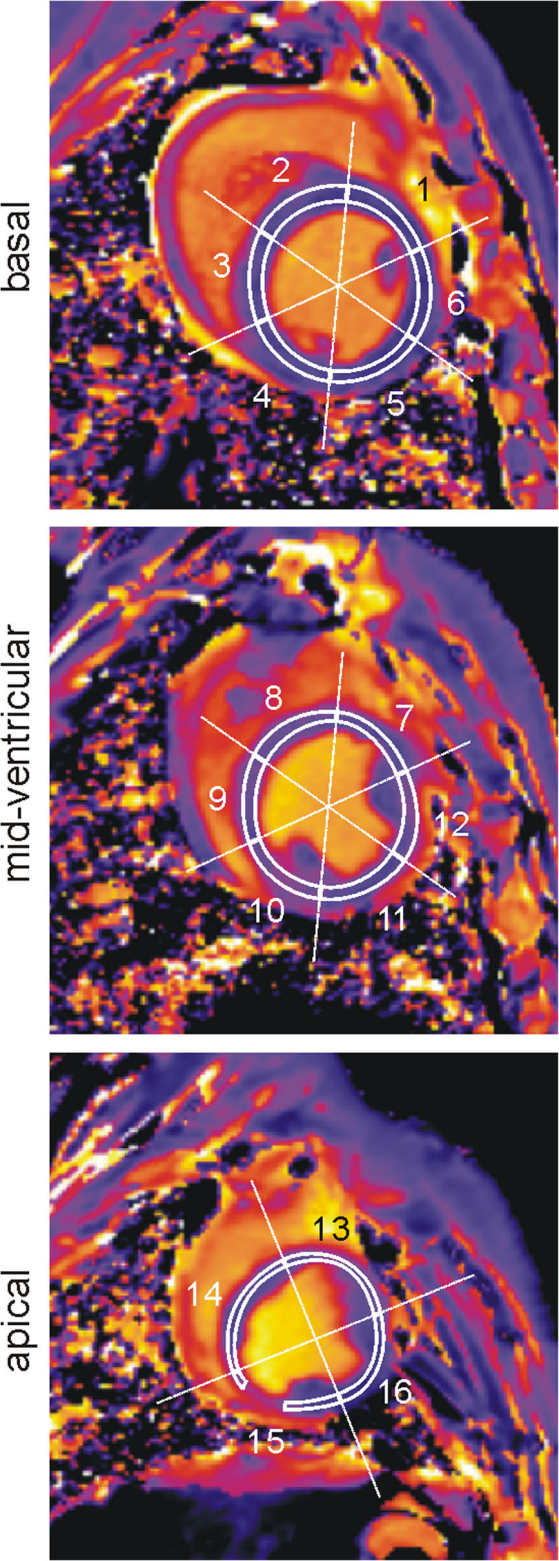
Native myocardial T1 maps. Regions for assessment of segmental myocardial T1 times (*white lines*) are overlaid. Segment 15 typically showed a susceptibility artefact from a prominent coronary vein which was carefully excluded from evaluation

An ECG-gated modified Look-Locker inversion recovery (MOLLI) prototype sequence with single-shot balanced steady-state free precession (bSSFP) readout, motion correction and automatic T1 map generation (MOLLI protocol 5(5)5(5)5; echo spacing, 2.6 ms; echo time, 1.1 ms; flip angle, 35°; voxel size, 2.1 × 1.4 × 8.0 mm^3^) was used to acquire myocardial T1 maps in end-diastole (Fig. 3).

### Left ventricular and myocardial function

Short-axis cine images were analyzed by syngo.via software (MR Cardiac Function, Siemens Healthcare, Erlangen, Germany). To assess LV volume vs. time curves, LV epicardial and endocardial borders, excluding papillary muscles from myocardium, were traced manually in end-diastole and end-systole and semi-automatically adjusted to all cardiac phases (Fig. 1). To define the basal plane, the position of the mitral valve was evaluated from the cine four-chamber view.

Normalized end-diastolic volume (EDV), end-systolic volume (ESV), stroke volume, cardiac output, and LV ejection fraction were evaluated from end-diastolic and end-systolic cardiac phases with the body surface area (BSA) estimated according to BSA (m^2^) = 0.0734 × weight (kg)^0.656^ [23]. The duration of LV diastasis (t_diastasis_) was derived from LV volume vs. time curves as the time interval starting after rapid LV filling and atrial contraction. Derivative of LV volume-time curves provided assessment of normalized peak ejection rate (PER), peak ejection time (PET), as well as early (PFR_E_, PFT_E_) and active (PFR_A_, PFT_A_) normalized peak filling rates and times (Fig. 1).

Papillary muscles were included when measuring normalized left ventricular mass (LVMM), but they were excluded in determining normalized mean end-diastolic and end-systolic wall thickness and thickening at the basal, mid-myocardial and apical levels; 16 myocardial segments were evaluated, according to the American Heart Association (AHA) segmentation scheme [24]. Global normalized end-diastolic (WT_ED_) and end-systolic (WT_ES_) wall thickness, as well as LV wall thickening, were calculated as averages of segmental values.

Times of mitral valve opening (MVO), mitral valve closure (MVC), and aortic valve opening (AVO)/closure (AVC) were derived from cine 3-chamber images by visual analysis (Fig. 1); in turn, these measurements were used to assess LV isovolumetric contraction (IVCT = AVO-MVC), isovolumetric relaxation (IVRT = MVO-AVC) and ejection (LVET = AVC-AVO) times. Index of myocardial performance (IMP) was calculated from IMP = (IVCT + IVRT)/LVET [25].

Mitral annular plane systolic excursion (MAPSE) was measured as the difference between the end-diastolic and the end-systolic distance from apex to lateral mitral annulus in 4-chamber view (Fig. 1).

### Left atrial volumes

Left atrial (LA) volumes were evaluated from manually tracing the LA area and length in 4- and 2-chamber views (Fig. 1). Normalized LA volumes were determined from cardiac phases before mitral valve opening at LV end-systole (maximum volume LAV_max_) and before mitral valve closure after LA contraction (minimum volume LAV_min_). For consistency, the plane of the mitral annulus was defined as the LA inferior border. Areas of recesses of the mitral valve, pulmonary veins and left atrial appendage were excluded by drawing a straight line across these structures to adjacent atrial borders. LA length was measured from the center of the mitral plane to the superior margin of the LA in 4- and 2-chamber views. Normalized LA volumes were estimated using the bi-planar area-length method [26] from LAV = 0.85 × A_2CH_ × A_4CH_/(L × BSA), where A_2CH_ and A_4CH_ are the LA areas in 2-chamber and 4-chamber views, respectively, and L is the length of the LA from either the 2- or the 4-chamber view (whichever is shorter). LA total ejection fraction (LATEF) was calculated according to LATEF = 100 × (LAV_max_-LAV_min_)/LAV_max_.

### Phase-contrast imaging data evaluation

Aortic peak velocity and cross-sectional area were evaluated from through-plane phase-contrast images by syngo.via (MR Flow Analysis, Siemens Healthcare, Erlangen, Germany). Aortic vessel wall thickness (AWT) was assessed from aortic cross section diameter measured from outer and inner vessel borders at the cardiac phases with maximum vessel cross section area at rest according to AWT = (outer diameter – inner diameter)/2.

**Fig. 4:**
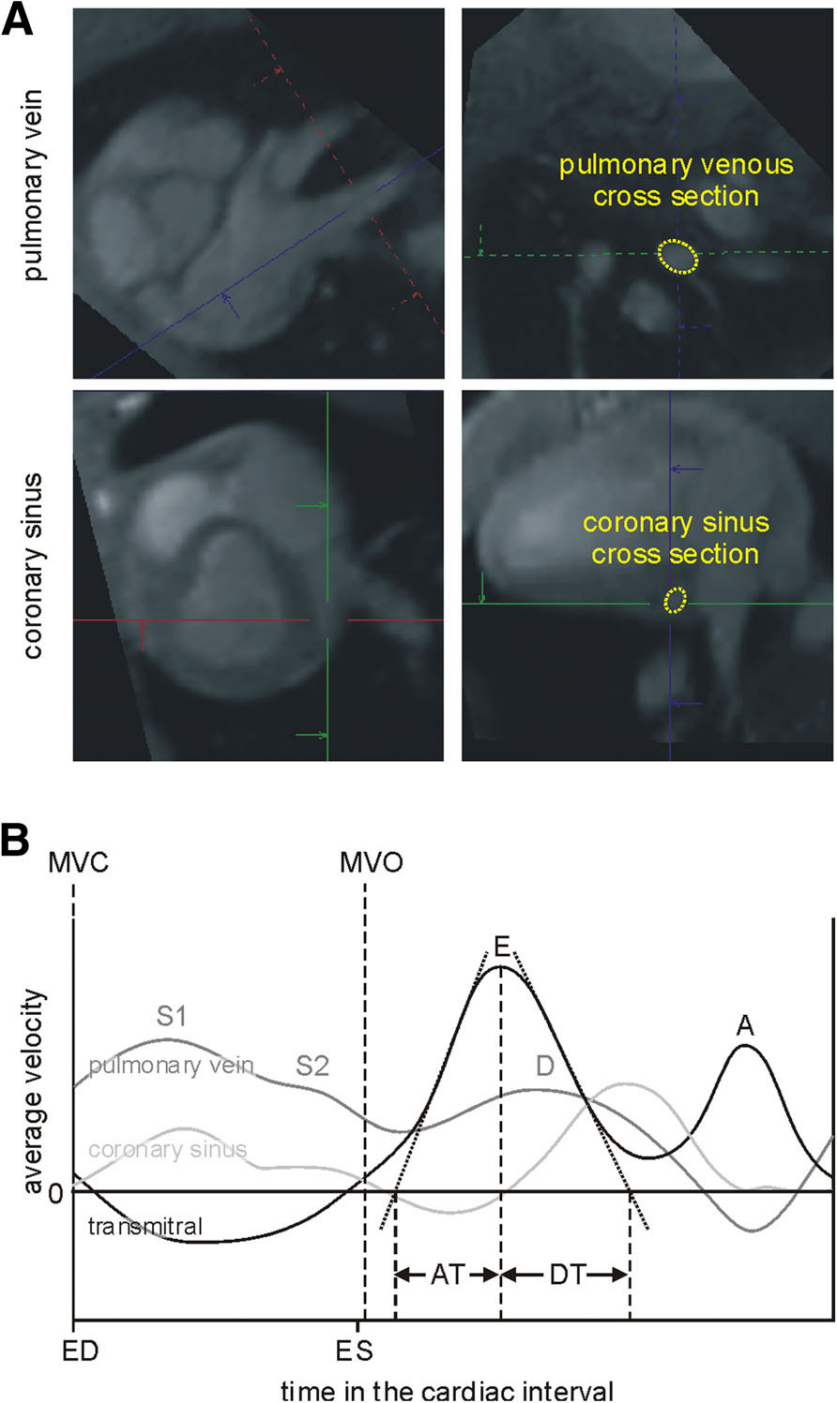
Evaluation of mitral, pulmonary venous and coronary sinus blood flow parameters from 4D flow data. **a** Cross-sectional areas were defined in multi-planar reformatted image planes of the anatomical phase-contrast data. The definition of pulmonary venous and coronary sinus cross sections are displayed. **b** Derived transmitral (*black line*), pulmonary venous (*gray line*), and coronary sinus (*light gray line*) average velocity vs. time courses. Peak through-plane velocities of transmitral early (E) and late (A) diastolic, pulmonary venous systolic (S1, S2) and early diastolic (D) were evaluated from respective peak velocity vs. time curves. E-wave acceleration (AT) and deceleration (DT) times were estimated by linear approximation of the acceleration and deceleration phase of the early diastolic transmitral average velocities. Coronary sinus net forwards blood volume was assessed from integration of average velocity × cross-sectional area. ED = time of LV end-diastole; ES = time of LV end-systole; MVC = time of mitral valve closure; MVO = time of mitral valve opening

Transmitral early (E) and late (A) diastolic, pulmonary venous systolic (S1, S2) and early diastolic (D) velocities, coronary sinus net forward blood flow volume (Fig. 4), and early diastolic lateral, septal and mean (E’) mitral annular tissue velocities were evaluated from multi-planar images reconstructed from 4D flow data using prototype software (4D Flow, Siemens Healthcare, Erlangen, Germany). Transmitral acceleration (AT) and deceleration (DT) times were assessed from average mitral velocity vs. time curves. E/A, E/E’, (E/E’)/EDV, and pulmonary venous S/D (with S as maximum of S1 and S2) ratios were calculated from peak through-plane velocities.

### Evaluation of global myocardial perfusion and perfusion reserve

Global myocardial perfusion (GMP) was derived as quotient of coronary sinus net forward blood flow volume multiplied by heart rate (which represents the time-averaged coronary sinus blood flow) and the left ventricular muscle mass [27]. Myocardial perfusion reserve (MPR) was calculated from stress-to-rest GMP [27].

### Evaluation of myocardial strain

Tagged images were evaluated semi-automatically by using prototype software (Heart Deformation Analysis 2.0, Siemens Healthcare, Erlangen, Germany and University of Auckland). A grid was aligned to the myocardial tags at end-diastole and propagated throughout all images of the cardiac cycle (Fig. 2). Grids were manually corrected to the tags if necessary. Myocardial circumferential (CC) and radial strain (RR) and strain rates (CC rate, RR rate) were calculated by the software from the motion of grid lines at the basal, mid-ventricular and apical levels from the respective short axis slices. Longitudinal myocardial strain (LL) and strain rates (LL rate) were assessed from 4-chamber view images, while myocardial torsion and torsion rates were assessed from the strains of basal and apical short-axis slices (Fig. 2). From time courses of strains, torsion and corresponding rates, end-systolic maxima/minima of strains and torsion as well as systolic and early diastolic maxima/minima of rates were determined. Furthermore, LV circumferential and radial strains and strain rates were calculated as means of basal, mid-ventricular and apical values.

### Evaluation of native myocardial T1 times

Segmental LV myocardial T1 times were derived by manually outlining T1 maps according to the AHA segmentation scheme, excluding blood pool, papillary muscles, trabeculae and epicardial structures. Regions were drawn to be as large as possible while avoiding inclusion of subendocardial and subepicardial tissue boundaries (Fig. 3). Global LV myocardial T1 was calculated as mean of segmental values.

### Electron microscopy and stereology

After in vivo measurements were completed, thoracotomy was performed and a bolus of 100 mmol potassium was administered intracoronarily to sacrifice each animal. For electron microscopic analysis of the myocardium, transmural biopsies were then collected from the lateral left ventricular wall and fixed in 1.5 % glutaraldehyde, 1.5 % paraformaldehyde in 0.15 M Hepes buffer. The samples were then stored in the fixative at 4 °C until further processing. The processing steps included post-fixation in 1 % osmium tetroxide solution, overnight staining in half-saturated uranyl acetate solution, dehydration in an ascending acetone series and finally embedding in epoxy resin. From the embedded samples, semi- and ultrathin sections were obtained for stereological analysis. The following parameters were assessed by the method of point counting [28]: volume fraction of interstitium (V_V_(int/lv)), volume fraction of cardiomyocytes (V_V_(myo,lv) = 1- V_V_(int/lv)), of blood vessels (V_V_(ves/lv)) and of collagen fibrils either related to the left ventricle (V_V_(coll/lv)) or to the interstitium (V_V_(coll/int)) as reference volume. The total myocyte (V(myo,lv)), collagen V(coll,lv) and blood vessel V(ves,lv) content in the myocardium were obtained by multiplying LVMM with V_V_(myo/lv), V_V_(coll/lv), and V_V_(ves/lv), respectively.

### Statistical analysis

Mean values are given together with standard deviations. Statistical analysis was performed using NCSS (Hintze, J. (2008) NCSS, LLC. Kaysville, Utah). A significance level of 0.05 was employed for statistical tests. Rest and stress indices of left heart and myocardial function as well as morphological and stereological parameters assessed in DOCA and control animals were compared by 2-sample *t*-test. Significances of differences of rest and stress indices or absolute and relative stress reserves were tested by 1-sample *t*-test.

## Results

DOCA pigs developed hypertension within 12 weeks. At rest, arterial blood pressure (mean, systolic and diastolic) and the rate-pressure product were higher in DOCA pigs than in controls; the groups did not differ significantly in weight, BSA and heart rate (Table 1). A stress-induced heart rate increase of 25 % was reached at significantly higher infusion rates in DOCA subjects (controls, 2.7 ± 0.7 μg · kg^−1^ · min^−1^; DOCA, 3.8 ± 1.0 μg · kg^−1^ · min^−1^). During stress, blood pressure in DOCA subjects failed to increase, and thus differences in blood pressures and rate-pressure product disappeared between groups.

### Left ventricular and myocardial function, left atrial volumetry

LVMM was higher in DOCA than in control animals (Table 2). DOCA animals revealed concentric hypertrophy with increased WT_ED_ and WT_ES_ at rest and during stress. LV wall thickening and MAPSE were different between groups at rest but not during stress.

**Table 2:** Parameters of cardiac and myocardial geometry and function

	Controls (*n* = 7)			DOCA (*n* = 5)			Controls vs. DOCA	
Parameter	Rest	Stress	*p*	Rest	Stress	*p*	p_rest_	p_stress_
LVMM (g · m^−2^)	84 ± 7			111 ± 9			<0.001	
WT_ED_ (mm · m^−2^)	6.3 ± 1.2	6.2 ± 1.4	0.934	8.3 ± 0.8	9.3 ± 1.6	0.224	0.007	0.006
WT_ES_ (mm · m^−2^)	9.6 ± 1.6	11.8 ± 2.2	0.009	13.0 ± 1.4	15.5 ± 1.9	0.012	0.003	0.012
WTH (mm · m^−2^)	3.3 ± 0.8	5.6 ± 1.7	0.019	4.8 ± 0.9	6.2 ± 1.1	0.074	0.020	0.489
MAPSE (mm)	11.1 ± 3.6	15.0 ± 3.7	0.078	6.9 ± 1.2	13.5 ± 4.9	0.040	0.037	0.586
EF (%)	52 ± 3	69 ± 7	0.001	53 ± 5	70 ± 2	< 0.001	0.840	0.749
EDV (ml · m^−2^)	124 ± 15	117 ± 12	0.293	125 ± 18	102 ± 26	0.008	0.862	0.203
ESV (ml · m^−2^)	59 ± 9	36 ± 10	0.005	59 ± 10	30 ± 9	< 0.001	0.983	0.360
SV (ml · m^−2^)	65 ± 8	81 ± 9	0.003	66 ± 11	71 ± 17	0.352	0.781	0.244
CI (l min^−1^ · m^−2^)	5.8 ± 0.9	9.4 ± 1.1	< 0.001	5.8 ± 1.0	7.8 ± 1.2	0.002	0.890	0.041
PER (ml · s^−1^ · m^−2^)	−380 ± 54	−711 ± 219	0.005	−419 ± 95	−639 ± 114	0.002	0.389	0.523
PFR_E_ (ml · s^−1^ · m^−2^)	307 ± 41	470 ± 75	0.002	332 ± 69	471 ± 49	0.007	0.449	0.979
PFR_A_ (ml · s^−1^ · m^−2^)	302 ± 73	345 ± 99	0.223	334 ± 133	277 ± 118	0.192	0.599	0.304
LAV_max_ (ml · m^−2^)	34 ± 4	32 ± 8	0.444	48 ± 17	34 ± 14	0.012	0.042	0.798
LAV_min_ (ml · m^−2^)	14 ± 3	14 ± 4	0.655	25 ± 9	21 ± 8	0.222	0.012	0.054
LATEF (%)	58 ± 4	58 ± 11	0.990	48 ± 14	36 ± 10	0.265	0.104	0.006

*LVMM* left ventricular muscle mass, *WT*_*ED*_ end-diastolic wall thickness, *WT*_*ES*_ end-systolic wall thickness, *WTH* wall thickening, *MAPSE* mitral annular plane systolic excursion, *EF* left ventricular ejection fraction, *EDV* left ventricular end-diastolic volume, *ESV* left ventricular end-systolic volume, *SV* left ventricular stroke volume, *CI* left ventricular cardiac index, *PER* peak ejection rate, *PFR*_*E*_ early diastolic peak filling rate, *PFR*_*A*_ late diastolic peak filling rate, *LAV*_*max*_ left atrial maximum volume, *LAV*_*min*_ left atrial minimal volume, *LATEF* left atrial total ejection fraction

*p* is related to the rest-stress comparison within each group. p_rest_ and p_stress_ relate to group comparisons at rest and stress, respectively

Stress-induced changes of systolic LV function indices were comparable for DOCA and control animals except for the increase in cardiac index, which was lower in DOCA animals (controls, 63 ± 23 %; DOCA, 37 ± 12 %; *p* = 0.040) and the decrease in EDV, which was higher in DOCA animals (controls, −5 ± 11 %; DOCA, −20 ± 11 %; *p* = 0.043). IVRT was longer in DOCA at rest but did not differ significantly between groups during stress (Table 3).

**Table 3:** Time intervals and indices of left ventricular function

	Controls (*n* = 7)			DOCA (*n* = 5)			Controls vs. DOCA	
Parameter	Rest	Stress	*p*	Rest	Stress	*p*	p_rest_	p_stress_
PET (ms)	82 ± 24	46 ± 23	0.006	110 ± 19	46 ± 16	0.007	0.053	0.972
PFT_E_ (ms)	382 ± 35	286 ± 19	< 0.001	389 ± 37	289 ± 34	0.001	0.752	0.831
PFT_A_ (ms)	586 ± 36	446 ± 13	< 0.001	622 ± 66	485 ± 67	0.007	0.255	0.160
IVRT (ms)	80 ± 15	78 ± 16	0.786	99 ± 10	93 ± 17	0.618	0.034	0.146
IVCT (ms)	46 ± 14	28 ± 8	0.013	56 ± 9	34 ± 11	< 0.001	0.175	0.330
LVET (ms)	205 ± 36	141 ± 22	0.008	194 ± 29	129 ± 53	0.005	0.600	0.606
IMP	0.64 ± 0.23	0.77 ± 0.18	0.353	0.82 ± 0.17	1.20 ± 0.82	0.313	0.179	0.201
t_diastase_ (ms)	90 ± 34	55 ± 28	0.017	94 ± 43	67 ± 14	0.139	0.858	0.408

*PET* peak ejection time, *PFT*_*E*_ early diastolic peak filling time, *PFT*_*A*_ late diastolic peak filling time, *IVRT* isovolumetric relaxation time, *IVCR* isovolumetric contraction time, *LVET* left ventricular ejection time, *IMP* index of myocardial performance, *t*_*diastase*_ duration of left ventricular diastasis

*p* is related to the rest-stress comparison within each group. p_rest_ and p_stress_ relate to group comparisons at rest and stress, respectively

Minimal and maximal LA volumes were enlarged in DOCA animals at rest. As maximal LA volume in the DOCA group significantly decreased during stress, LATEF, which was comparable between groups at rest, was significantly smaller in the DOCA group during stress (Table 2).

### Phase-contrast velocity mapping

Aortic wall was thicker in DOCA pigs (Table 4). Aortic blood peak velocity was higher in controls than in DOCA animals at rest but equalized during stress. At rest, the minimal, maximal and average aortic cross-sectional areas were larger in DOCA than in control pigs; with stress, these differences became insignificant except for the difference in minimal aortic cross-sectional area.

**Table 4:** Phase-contrast velocity mapping-based parameters

	Controls (*n* = 7)			DOCA (*n* = 5)			Controls vs. DOCA	
Parameter	Rest	Stress	*p*	Rest	Stress	*p*	p_rest_	p_stress_
Aortic vessel wall and aortic blood velocity								
APV (cm · s^−1^)	113 ± 15	158 ± 26	0.001	92 ± 14	140 ± 31	0.011	0.033	0.308
AA_mean_ (mm^−2^)	3.4 ± 0.4	3.7 ± 0.7	0.091	5.5 ± 0.5	5.0 ± 1.3	0.244	< 0.001	0.064
AA_min_ (mm^−2^)	2.8 ± 0.4	3.0 ± 0.6	0.211	4.6 ± 0.5	4.2 ± 1.1	0.229	< 0.001	0.044
AA_max_ (mm^−2^)	4.1 ± 0.4	4.5 ± 0.6	0.061	6.3 ± 0.5	5.7 ± 1.4	0.228	< 0.001	0.072
AWT (mm)	1.8 ± 0.3			2.3 ± 0.4			0.039	
Transmitral and myocardial tissue velocities								
E (cm · s^−1^)	63 ± 6	78 ± 9	0.001	56 ± 10	70 ± 11	0.001	0.181	0.222
A (cm · s^−1^)	47 ± 11	48 ± 9	0.714	43 ± 12	43 ± 13	0.801	0.532	0.404
E/A	1.4 ± 0.5	1.7 ± 0.5	0.100	1.4 ± 0.4	1.7 ± 0.5	< 0.001	0.861	0.886
AT (ms)	87 ± 17	79 ± 9	0.329	83 ± 14	70 ± 9	0.091	0.639	0.120
DT (ms)	152 ± 50	163 ± 66	0.705	169 ± 67	153 ± 16	0.639	0.622	0.760
E’_sep_ (cm · s^−1^)	18 ± 4	20 ± 6	0.743	11 ± 3	14 ± 2	0.109	0.010	0.069
E’_lat_ (cm · s^−1^)	21 ± 5	25 ± 6	0.346	14 ± 3	16 ± 3	0.213	0.067	0.019
E’ (cm · s^−1^)	20 ± 4	23 ± 6	0.492	13 ± 2	15 ± 2	0.130	0.007	0.025
E/E’	3.3 ± 0.6	3.6 ± 0.7	0.185	4.4 ± 1.1	4.6 ± 0.6	0.707	0.038	0.031
(E/E’)/EDV (10^−2^ ml^−1^ · m^2^)	2.7 ± 0.5	3.1 ± 0.6	0.151	3.6 ± 0.9	4.8 ± 1.6	0.112	0.035	0.030
Pulmonary venous velocities								
S1 (cm · s^−1^)	33 ± 9	48 ± 4	0.006	39 ± 18	46 ± 5	0.516	0.502	0.408
S2 (cm · s^−1^)	40 ± 10	49 ± 9	0.032	44 ± 18	44 ± 5	0.668	0.624	0.484
D (cm · s^−1^)	37 ± 10	56 ± 11	< 0.001	57 ± 17	58 ± 6	0.792	0.035	0.636
S/D	1.1 ± 0.2	0.9 ± 0.2	0.019	0.8 ± 0.2	0.8 ± 0.2	0.864	0.045	0.194
Coronary sinus								
NFV_CS_ (ml · m^−2^)	1.6 ± 0.5	2.7 ± 1.0	0.003	2.4 ± 0.6	2.8 ± 0.5	0.300	0.025	0.886
GMP (ml · min^−1^ · g^−1^)	1.6 ± 0.4	3.7 ± 1.4	0.002	1.9 ± 0.3	2.9 ± 0.5	0.029	0.197	0.268
MPR		2.2 ± 0.5			1.5 ± 0.4			0.028

*APV* aortic peak velocity, *AA*_*mean*_ average aortic cross section area, *AA*_*min*_ minimal aortic cross section area, *AA*_*max*_ maximal aortic cross section area, *AWT* aortic wall thickness, *E* early diastolic transmitral peak velocity, *A* late diastolic transmitral peak velocity, *E’*_*sept*_ early diastolic septal wall mitral annular tissue velocity, *E’*_*lat*_ early diastolic lateral wall mitral annular tissue velocity, *E’* average early diastolic mitral annular tissue velocity, *S1* early systolic pulmonary venous peak velocity, *S2* systolic pulmonary venous peak velocity, *D* early diastolic pulmonary venous peak velocity, *S* maximal systolic pulmonary venous peak velocity, *NFV*_*CS*_ coronary sinus net forward volume, *GMP* global myocardial perfusion, *MPR* stress-to-rest myocardial perfusion reserve

*p* is related to the rest-stress comparison within each group. p_rest_ and p_stress_ relate to group comparisons at rest and stress, respectively

Early diastolic myocardial tissue peak velocity values were lower in DOCA pigs and corresponded to higher E/E’ in the DOCA group, even though transmitral inflow patterns did not differ between groups (Table 4). (E/E′)/EDV was higher in DOCA pigs both at rest and during stress.

The main differences in the pulmonary venous flow patterns between the two groups were that DOCA pigs showed significantly higher D-wave velocities at rest, while controls displayed a significant stress-induced increase in pulmonary venous peak velocities that was not observed in the DOCA group. Accordingly, the S/D ratio differed significantly between the two groups at rest but not during stress.

### Global myocardial perfusion and perfusion reserve

Under rest coronary sinus net forward blood volume was larger in the DOCA pigs than in the control pigs, but equalized under stress condition. Although global myocardial perfusion did not differ significantly between groups and increased with stress in both groups, myocardial perfusion reserve was significantly smaller in the DOCA group (Table 4).

### LV myocardial strains and torsion

Circumferential, radial and longitudinal strains and strain rates as well as torsion and torsion rates were evaluated in all DOCA and 6 controls pigs. One control pig was excluded from evaluation due to poor tagged imaging quality.

Differences between DOCA and control pigs were found in the longitudinal strain and torsion rates (Table 5). Whereas systolic LL rate_min_ did not differ between groups at rest, it was smaller in DOCA animals during stress. Systolic torsion rate_max_ showed the opposite behavior, being significantly higher in DOCA pigs at rest but not during stress. Diastolic torsion rate_min_ did differ significantly between groups; however, the torsion rate_min_ failed to properly increase during stress in the DOCA group (control, −19 ± 11° · s^−1^; DOCA, 0 ± 5° · s^−1^; *p* = 0.015).

**Table 5:** Left ventricular myocardial strain and torsion

	Controls (*n* = 6)			DOCA (*n* = 5)			Controls vs. DOCA	
Parameter	Rest	Stress	*p*	Rest	Stress	*p*	p_rest_	p_stress_
CC_min,LV_ (%)	−16 ± 1	−17 ± 2	0.887	−17 ± 2	−18 ± 3	0.404	0.728	0.284
RR_max,LV_ (%)	34 ± 12	39 ± 11	0.163	33 ± 4	35 ± 5	0.533	0.764	0.481
LL_min,LV_ (%)	−13 ± 2	−13 ± 3	0.495	−12 ± 1	−11 ± 2	0.342	0.187	0.337
torsion_max_ (°)	4.6 ± 1.4	5.7 ± 1.6	0.560	6.1 ± 0.9	7.0 ± 1.2	0.061	0.098	0.231
CC rate_min,LV_ (% · s^−1^)	−90 ± 11	−139 ± 19	0.009	−96 ± 13	−155 ± 16	< 0.001	0.436	0.182
RR rate_max,LV_ (% · s^−1^)	185 ± 52	337 ± 66	< 0.001	188 ± 36	309 ± 81	0.015	0.908	0.540
LL rate_min_ (% · s^−1^)	−77 ± 11	−120 ± 15	0.002	−68 ± 6	−95 ± 13	0.004	0.120	0.024
torsion rate_max_ (° · s^−1^)	29 ± 3	47 ± 15	0.044	44 ± 8	62 ± 17	0.103	0.002	0.193
CC rate_max,LV_ (% · s^−1^)	83 ± 4	119 ± 30	0.023	89 ± 11	129 ± 20	0.005	0.240	0.542
RR rate_min,LV_ (% s^−1^)	−190 ± 81	−229 ± 90	0.132	−192 ± 41	−222 ± 53	0.348	0.956	0.881
LL rate_max_ (% · s^−1^)	51 ± 19	71 ± 17	0.104	47 ± 10	58 ± 10	0.143	0.670	0.171
torsion rate_min_ (° · s^−1^)	−24 ± 6	−43 ± 13	0.009	−28 ± 9	−28 ± 7	0.921	0.457	0.072

*CC*_*min,LV*_ minimal left ventricular circumferencial strain, *RR*_*max,LV*_ maximal left ventricular radial strain, *LL*_*min,LV*_ minimal left ventricular longitudinal strain, *torsion*_*max*_ maximal left ventricular torsion, *CC rate*_*min,LV*_ systolic minimum circumferencial strain rate, *RR rate*_*max,LV*_ systolic maximal radial strain rate, *LL rate*_*min*_ systolic minimal longitudinal strain rate, *torsion rate*_*min*_ systolic minimal torsion rate, *CC rate*_*max,LV*_ early diastolic maximal circumferencial strain rate, *RR rate*_*min,LV*_ early diastolic minimal radial strain rate, *LL rate*_*max*_ early diastolic maximal longitudinal strain rate, *torsion rate*_*max*_ early diastolic maximal torsion rate

*p* is related to the rest-stress comparison within each group. p_rest_ and p_stress_ relate to group comparisons at rest and stress, respectively

### Global LV myocardial T1 times

Native myocardial T1 relaxation times were evaluated in 5 DOCA and 6 control animals. One control pig was excluded from evaluation due to poor image quality. No differences between groups were found in global native T1 times (controls, 1195 ± 36 ms; DOCA, 1161 ± 21 ms; *p* = 0.094).

### Stereological analysis

**Fig. 5:**
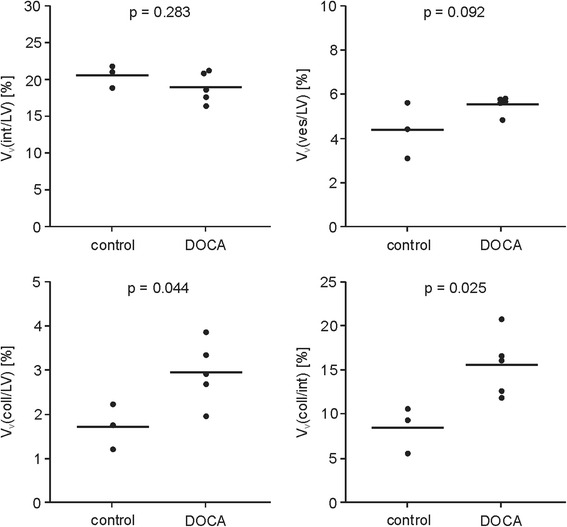
Dot plots of volume fractions of the interstitium V_V_(int/lv), of the blood vessels V_V_(ves/lv), and of collagen V_V_(coll/lv) with respect to the LV myocardium, as well as volume fraction of collagen with respect to the interstitium V_V_(coll/int) in controls and DOCA pigs. Lines indicate mean values, *p* values refer to *t*-test

Samples of five DOCA and three control pigs were evaluated. Volume fractions of the interstitium (V_V_(int/lv): controls, 21 ± 2 %; DOCA, 19 ± 2 %; *p* = 0.283) and of blood vessels (V_V_(ves/lv): controls, 4.4 ± 1.3 %; DOCA, 5.5 ± 0.4 %; *p* = 0.092) did not differ between the DOCA-salt treated and control group. Volume fractions of collagen with respect to both the LV (V_V_(coll/lv): controls, 1.7 ± 0.5 %; DOCA, 3.0 ± 0.7 %; *p* = 0.044) and the interstitium (V_V_(coll/int): controls, 8 ± 3 %; DOCA, 16 ± 4 %; *p* = 0.025) were significantly increased in the DOCA group (Fig. 5). Total myocyte volume (V(myo/lv): controls, 67 ± 1 ml; DOCA, 104 ± 16 ml; *p* = 0.009), total collagen volume (V(col/lv): controls, 1.5 ± 0.4 ml; DOCA, 3.8 ± 1.0 ml; *p* = 0.011) and total blood vessel volume (V(ves/lv): controls, 3.7 ± 1.1 ml; DOCA, 7.1 ± 1.2 ml; *p* = 0.012) were higher in the DOCA pigs than in the control pigs.

## Discussion

CMR at rest and during dobutamine stress allowed analysis and characterization of cardiac, cardiovascular and myocardial function in pigs without and with DOCA-salt treatment. Aside from LV hypertrophy, DOCA animals displayed alterations in myocardial muscle mechanics, left ventricular filling characteristics, left atrial volumes and function, and myocardial perfusion at rest and/or during stress. Significant elevation of collagen in the DOCA group shown by stereology could not be resolved by native T1 mapping.

### Left ventricular hypertrophy and myocardial alterations

In accordance with previous studies, DOCA pigs developed hypertension and concentric LV hypertrophy, with a normal-sized LV chamber and increased LV muscle mass [9, 10, 17]. Stereological interstitial volume fraction was comparable in DOCA and control animals, indicating that hypertrophy in this early-stage HFpEF model results from enlargement of both cardiomyocytes and interstitial space. In the study by Schwarzl et al. [17], picrosirius red staining did not indicate higher levels of collagen in DOCA pigs compared to control animals. Stereological analysis in the present study revealed increased levels of collagen (i.e., a higher collagen volume relative to the interstitial space, relative to the LV myocardium and to the absolute volume). Notably, increased levels of collagen and reduced total-titin phosphorylation [17] in DOCA pigs could be interpreted as a sign of a transition from hypertensive heart disease to HFpEF [29–32].

Native T1 times did not resolve the higher myocardial collagen content documented by the stereological evaluation [33, 34]. This can be understood by the fact that voxel-based evaluation of magnetic relaxation time maps displays an effective T1 time of all compartments in the voxel. An increased LV volume fraction of collagen tends to increase myocardial T1 times [33], whereas an increased volume fraction of cardiomyocytes should decrease T1. Even though not significant, lower native myocardial T1 times in early-stage HFpEF animals represent the larger compartment (1- V_V_(int/lv) in stereological analysis) of hypertrophied cardiomyocytes rather than the elevated levels of collagen in the slightly smaller compartment of interstitium (V_V_(int/lv) in stereological analysis) in a voxel.

### Blood supply and myocardial perfusion

In accordance with other investigations of vessel wall thickening in DOCA pigs [12, 14], our study found significantly enlarged aortic cross-sectional areas and increased aortic vessel wall thickness in the DOCA group. In addition to this aortic remodeling, the absolute myocardial vessel volume compartment V(ves/lv) was enlarged in DOCA pigs, which could be due to the increase of vessel wall thickness, number of coronary vessels, coronary vessel dilatation, or all together; microvascular rarefaction as found in HFpEF patients at higher levels of myocardial fibrosis [35] is, however, not directly supported.

Although the exact cause for the altered myocardial vessel volume compartment in DOCA pigs cannot be specified on the basis of stereological data, structural changes in the myocardial microvasculature are in line with the impairment of the myocardial perfusion reserve observed in DOCA pigs: Coronary sinus net forward volume was higher in DOCA pigs at rest maintaining normal global myocardial perfusion of the hypertrophic myocardium. During stress, myocardial perfusion in DOCA pigs increased mainly due to a rise in heart rate (indicated by the insignificant stress-to-rest difference of coronary sinus net forward volume), resulting in a significantly reduced myocardial perfusion reserve in DOCA pigs compared to controls. These findings at rest and stress were also reported in patients with HFpEF [36] and were attributed to microvascular dysfunction as well as a reduced vasodilator reserve [36, 37].

### Left ventricular and myocardial function

During β-adrenergic stress, an adequate increase in LV ejection fraction and a decrease in ESV accounted for normal contractility reserves in both groups. However, EDV significantly decreased during stress in DOCA pigs, inhibiting stroke volume and cardiac index from properly increasing with the heart rate. A similar response was reported in patients with HFpEF during dynamic exercise [38, 39].

Echocardiographic studies in HFpEF patients document reduced longitudinal and circumferential strain [40] as well as failure to increase LV ejection fraction and global longitudinal strain rate during stress [41]. In our study, DOCA pigs with early-stage HFpEF did not show failure to increase LV ejection fraction during stress, but they demonstrated a significantly lower LL rate_min_ compared to control animals; thus systolic function – though normal at rest – showed signs of impairment during stress. These results are in accordance with findings in hypertensive patients with LV hypertrophy, where authors showed that systolic dysfunction may develop in parallel to diastolic dysfunction [40, 42, 43]. Moreover and in line with findings in patients with hypertensive LV hypertrophy [44], MAPSE was reduced in the DOCA group at rest but significantly increased during stress enabling adequate increase in LV ejection fraction with heart rate.

E’ was significantly decreased in the DOCA group at rest and during dobutamine stress, and accordingly E/E’ and (E/E’)/EDV ratios were higher in DOCA than in control animals. It was previously shown in HFPEF patients that E/E’ correlates well with the LV end-diastolic pressure [39]. The significant higher E/E’ in DOCA compared to control pigs at rest and during stress indicates slightly higher LV filling pressures in DOCA animals, which is up to statistical significance in accordance with the invasive results reported by Schwarzl et al. [17]. Decreased E’ and increased (E/E’)/EDV might be interpreted as marker for increased diastolic myocardial stiffening in DOCA pigs [32], caused by both, increased levels of collagen shown in the present study and reduced total-titin phosphorylation reported by Schwarzl et al. [17]. Similar changes in E’ and (E/E’)/EDV were observed also in HFpEF patients [32, 39, 45]. In DOCA pigs, increased (E/E’)/EDV may further be related to the prolonged IVRT at rest [46, 47] and/or subtle stress-induced myocardial ischemia [48], as indicated by the lower global myocardial perfusion reserve during dobutamine in the hypertrophied LV myocardium in DOCA pigs.

Due to increased D-wave peak velocities in the DOCA group, the pulmonary venous S/D ratio was significantly lower at rest in DOCA pigs than in control pigs. As LV relaxation is the main determinant of pulmonary venous flow [49, 50], observed pulmonary venous flow patterns in DOCA pigs again indicate altered LV relaxation. The observed failing of systolic and diastolic pulmonary venous peak velocities to increase during stress in the DOCA group could be due to a mild LA pressure increase [17, 50], which could in turn be related to the increased E/E’ in DOCA animals.

LV torsion is known to be dependent on LV shape, and in LV concentric hypertrophy increased torsion is due to an increased lever arm for epicardial fibers [51]. Accordingly, torsion and systolic torsion rate_max_ were higher in DOCA than control pigs. Diastolic torsion rate_min_ significantly increased during β-adrenergic stimulation in the control group, indicating that intraventricular pressure gradients appropriately increased [52]. In DOCA animals, the difference in torsion rate_min_ between stress and rest failed to properly increase; this failure relates to reduced intraventricular pressure gradients and impairment of LV relaxation [53].

### Left atrium

DOCA pigs showed significantly increased LA volumes at rest; this could be interpreted as a marker of altered diastolic function, LA pressure and early diastolic filling [54]. During stress, maximal LA volumes significantly decreased in the DOCA group (in parallel with EDV), augmenting impairment of LATEF. Melenovsky et al. [45] found that among various systolic, diastolic, and vascular function abnormalities seen in patients with LV hypertrophy and patients with HFpEF, LA dilatation and reduced LATEF were the most useful for discriminating between the two groups.

### Limitations

Several limitations of the present study need to be acknowledged. The study had a small sample size; therefore it was not possible to assess correlations between studied parameters. Moreover, samples for stereological analysis were collected in a sub-group of animals only from the LV lateral wall and, for reasons of feasibility, not by a random sampling scheme.

Cardiac, myocardial and vascular CMR parameters were obtained for comparison from DOCA-treated and non-DOCA treated landrace pigs in anesthesia. As there were no obvious cardiovascular malformations and no outliers in the studied parameters, it is quite likely that all the non-DOCA treated pigs represent a normal collective. Neither the effect of the subcutaneous implantation procedure nor the overall effects of anesthesia were controlled in the current study.

All CMR measurements were performed under mechanical ventilation, which reduced limitations on temporal resolution of cine acquisitions, in particular. The fact that diastolic functional parameters like E’, E/A or IVRT compare well with echocardiographic normal values in pigs [55, 56] might be interpreted as sign for an adequate choice of temporal resolution of cine sequences to unmask possible differences in diastolic function of DOCA and control pigs. Breathing motion was typically suppressed by averaging, except for the MOLLI sequence. Automated motion correction, however, enabled appropriate reconstruction of T1 maps. 4D flow data were acquired with one velocity encoding optimized for LV intra-cavity blood flow, and all flow results were determined a posteriori from this dataset. The multiple acquisitions of smaller data sets with optimized velocity encoding and optimized resolution might have improved the accuracy of results but would have further prolonged investigation time.

The comprehensive imaging protocol allowed the investigation of only one stress level. In accordance with results found in HFpEF patients [57], chronotropic responsiveness to low-dose dobutamine was slightly reduced in DOCA pigs compared to controls, necessitating increased dobutamine infusion rates for DOCA pigs when targeting a heart rate increase of approximately 25 % in all subjects. While equalizing chronotropic responses during stress in DOCA and control pigs, dobutamine dosage and its inotropic, lusitropic and vasodilative effects were not controlled. Continuous and monotone responses of myocardial functional parameters found at small increases of infusion rates of dobutamine in the low-dose regime in HFpEF and control patients [57] suggest, that only small differences in observed stress-to-rest reserves might be expected compared to applying constant dobutamine infusion rates in all pigs.

Finally, invasive intra-cardiac hemodynamic measurements were not performed during CMR examinations, as appropriate MR-compatible equipment was not available.

## Conclusions

The present study documents numerous alterations in CMR-derived indices of cardiac and myocardial function at rest and during stress in pigs with DOCA-salt induced early-stage HFpEF. LA enlargement, metrics of myocardial tissue velocity, pulmonary venous and transmitral blood flow velocities presented as potential CMR markers of early-stage HFpEF at rest, highlighting the important role of LA impairment in the development of HFpEF. Inadequate increases in myocardial perfusion reserve and cardiac index during dobutamine stress may prove to be useful new CMR metrics for the diagnosis of HFpEF, and could probably account for exercise intolerance in early stages of disease. Myocardial T1 mapping, however, could not detect elevated levels of myocardial collagen found by stereology in DOCA pigs.

## Abbreviations

A: Late diastolic mitral peak blood flow velocity
AT: Acceleration time
AVC: Aortic valve closure
AVO: Aortic valve opening
AWT: Aortic wall thickness
BSA: Body surface area
bSSFP: Balanced steady-state free precession
CC: Circumferential strain
CMR: Cardiovascular magnetic resonance
D: Early diastolic pulmonary venous peak velocity
DT: Deceleration time
E: Early diastolic mitral peak blood flow velocity
E’: Early diastolic myocardial tissue velocity
ECG: Electrocardiographical
EDV: LV end-diastolic volume
ESV: LV end-systolic volume
FLASH: Fast low angle shot
HFpEF: Heart failure with preserved ejection fraction
IVCT: Isovolumetric contraction time
IVRT: Isovolumetric relaxation time
LA: Left atrium
LATEF: Total left atrial ejection fraction
LAV: LA volume
LL: Longitudinal strain
LV: Left ventricle
LVET: Left ventricular ejection time
LVMM: Left ventricular muscle mass
MAPSE: Mitral annular plane systolic excursion
MOLLI: Modified Look-Locker inversion recovery
MVC: Mitral valve closure
MVO: Mitral valve opening
PER: Peak ejection rate
PET: Peak ejection time
PFR_A_: Late diastolic peak filling rate
PFR_E_: Early diastolic peak filling rate
PFT_A_: Late diastolic peak filling time
PFT_E_: Early diastolic peak filling time
RR: Radial strain
S: Systolic pulmonary venous peak blood flow velocity
SPAMM: Spatial modulation of magnetization
t: time
V(coll,lv): Total myocardial collagen content
V(myo,lv): Total myocardial myocyte content
V(ves,lv): Total myocardial blood vessel content
V_V_(coll/int): Volume fraction of collagen fibrils related to the interstitium
V_V_(coll/lv): Volume fraction of collagen fibrils related to the left ventricle
V_V_(int/lv): Volume fraction of the interstitium related to the left ventricle
V_V_(myo,lv): Volume fraction of cardiomyocytes related to the left ventricle
V_V_(ves/lv): Volume fraction of blood vessels related to the left ventricle
